# Aryl Hydrocarbon Receptor-Mediated Disruption of Intestinal Epithelial Barrier Integrity by Dioxin Isomers

**DOI:** 10.3390/toxics13110993

**Published:** 2025-11-18

**Authors:** Hideki Kakutani, Teruyuki Nakao

**Affiliations:** Laboratory of Disease Prevention, Faculty of Pharmaceutical Sciences, Setsunan University, 45-1 Nagaotoge-cho, Hirakata 573-0101, Osaka, Japan

**Keywords:** AhR ligand, epithelial barrier function, tight junction

## Abstract

The intestinal epithelium constitutes a critical barrier that protects the host from luminal toxins. Persistent organic pollutants (POPs), including dioxins and dioxin-like polychlorinated biphenyls, are ubiquitous aryl hydrocarbon receptor (AhR) ligands. However, their effects on intestinal barrier integrity remain poorly understood. We examined representative POPs in vitro (using human Caco-2 monolayers) and in vivo (using a mouse jejunal loop model). Measurements of transepithelial electrical resistance, fluorescein isothiocyanate–dextran permeability, and cytotoxicity revealed that 2,3,7,8-tetrachlorodibenzo-*p*-dioxin (TCDD) impaired barrier function at non-cytotoxic concentrations. This effect was accompanied by increased ethoxyresorufin-*O*-deethylase activity and subsequently reversed by the AhR antagonist CH223191, indicating AhR dependence. Mechanistically, TCDD suppressed claudin-1, claudin-4, and zonula occludens-1 expression while upregulating the transcription factor Slug, consistent with junctional remodeling. In vivo, TCDD enhanced systemic dextran leakage and reduced claudin-4 expression in jejunal epithelia. These findings identify intestinal barrier disruption as a sensitive toxicological endpoint of POP exposure and provide mechanistic insight into the link between environmental pollutants and gastrointestinal dysfunction.

## 1. Introduction

The intestinal epithelium constitutes a highly dynamic interface that ensures efficient nutrient and water absorption while preventing the entry of luminal toxins, pathogens, and antigens. Tight junction (TJ) complexes—composed of claudins (CL), occludin, and zonula occludens (ZO) scaffolding proteins—serve as the critical structural and regulatory determinants of paracellular permeability [1,2]. Perturbations of epithelial barrier integrity are increasingly recognized as central events in the pathogenesis of inflammatory bowel disease, food allergies, irritable bowel syndrome, obesity-associated metabolic dysfunction, and extraintestinal conditions such as systemic inflammation and neurodegeneration [3,4,5,6].

Persistent organic pollutants (POPs), including polychlorinated dibenzo-*p*-dioxins and dibenzofurans, coplanar polychlorinated biphenyls (PCBs), and polycyclic aromatic hydrocarbons (PAHs), represent a major class of environmental contaminants characterized by chemical stability, bioaccumulative potential, and long half-lives [7,8]. Accumulating epidemiological evidence indicates that exposure to POPs contributes to an increased risk of immune dysfunction, metabolic disorders, and gastrointestinal diseases [9,10,11]. Despite this evidence, the direct effects of POPs on intestinal epithelial barrier integrity remain poorly defined.

The aryl hydrocarbon receptor (AhR), a ligand-activated transcription factor that regulates xenobiotic metabolism, immune function, and epithelial homeostasis, serves as a key mediator of the toxicological actions of numerous POPs [12,13]. Activation of AhR by high-affinity ligands such as 2,3,7,8-tetrachlorodibenzo-*p*-dioxin (TCDD) induces cytochrome P450 1A1 (CYP1A1) expression, a hallmark of AhR signaling [14]. Beyond xenobiotic metabolism, AhR signaling has emerged as a critical regulator of intestinal physiology, balancing protective and disruptive responses depending on the nature of its ligands [15]. For instance, dietary indoles and microbial tryptophan metabolites reinforce epithelial barrier integrity and maintain mucosal homeostasis [16,17], whereas persistent toxicants such as dioxins and dioxin-like PCBs reportedly impair gut barrier function and promote inflammation [18,19].

Despite this duality, mechanistic studies directly linking POP exposure, AhR activation, and intestinal barrier disruption remain limited. In particular, it is unclear whether pollutant-induced barrier dysfunction occurs independently of cytotoxicity, which specific junctional components are primarily affected, and whether these alterations are recapitulated in vivo. Addressing these knowledge gaps is essential for toxicological risk assessment, given the pervasive nature of POPs exposure in human populations.

In this study, we investigated the effects of representative environmental pollutants on intestinal barrier function using human Caco-2 monolayers, with a focus on the involvement of AhR signaling. Barrier integrity was evaluated through transepithelial electrical resistance (TEER), paracellular permeability measurements, and analysis of junctional protein expression. Furthermore, in situ mouse jejunal loop experiments were performed to assess the in vivo relevance of our findings. Ultimately, the results provide mechanistic insights into the impact of AhR ligands on intestinal barrier disruption and underscore their toxicological significance.

## 2. Material and Methods

### 2.1. Chemicals

2,3,7,8-TCDD, 1,3,6,8-TCDD, and other environmental pollutants were purchased from Cambridge Isotope Laboratories (Tewksbury, MA, USA). CH223192 was obtained from Sigma-Aldrich (St. Louis, MO, USA). All chemicals were dissolved in dimethyl sulfoxide (DMSO), and the final DMSO concentration in the culture medium was kept at 0.2% (*v*/*v*).

### 2.2. Cell Culture

Human intestinal Caco-2 cells (passages 25–30) were obtained from the American Type Culture Collection (Rockville, MD, USA) and maintained in Dulbecco’s modified Eagle’s medium (DMEM) supplemented with 10% fetal bovine serum and 1% nonessential amino acids at 37 °C in a 5% CO_2_ atmosphere. Cytotoxic effects of the tested chemicals were assessed using the WST-8 assay (Nacalai Tesque, Kyoto, Japan) and the CytoTox96 Non-Radioactive Cytotoxicity Assay (Promega, Madison, WI, USA) according to the manufacturers’ instructions.

### 2.3. Measurement of TEER

Caco-2 cells were seeded at a subconfluent density onto Transwell chambers (Corning, Corning, NY, USA) and cultured for 21 days to allow for complete differentiation. Transepithelial electrical resistance (TEER), an indicator of tight junction (TJ) integrity, was measured using a Millicell-ERS epithelial volt-ohmmeter (Merck Millipore, Darmstadt, Germany). Monolayers were considered ready for use once they reached stable plateau values, typically 800–1000 Ω·cm^2^, confirming the formation of intact monolayers prior to treatment. Cells were then treated apically with the test chemicals, and TEER values were subsequently recorded at defined time points. Background resistance from blank Transwell inserts was subtracted from all measured readings.

### 2.4. Ethoxyresorufin-O-Deethylase (EROD) Activity Assay

Caco-2 cells were plated at 5 × 10^4^ cells/well in 96-well microplates and incubated under the treatment conditions specified in the Results section. Following incubation, cells were treated with the substrate, 5 µM 7-ethoxyresorufin (Sigma-Aldrich) for 1 h at 37 °C. The fluorescence of resorufin was measured at 550 nm excitation and 595 nm emission using a TriStar LB 941 microplate reader (Berthold, Bad Wildbad, Germany). EROD activity was normalized to protein content, which was quantified by a BCA protein assay kit (Thermo Scientific, Rockford, IL, USA).

### 2.5. Paracellular Tracer Flux Assay

Caco-2 monolayers in Transwell chambers were exposed to the test chemicals for 48 h. Fluorescently labeled FITC-dextran (4–10 kDa; Sigma-Aldrich) or 4–10 kDa dextran (Sigma-Aldrich) was prepared in culture medium and applied to the apical or basolateral compartment at a final concentration of 5 mg/mL. Following 1 h of incubation, the fluorescence in the basolateral compartment was quantified using a TriStar LB 941 microplate reader at 485 nm excitation and 535 nm emission.

### 2.6. Measurement of TJ-Related Protein mRNA Expression Using Real-Time Reverse Transcription Polymerase Chain Reaction (RT-qPCR)

Total RNA was extracted from treated Caco-2 cells using RNAiso Plus (TaKaRa, Kyoto, Japan) and reverse-transcribed with PrimeScript RT Master Mix (TaKaRa). RT-qPCR was conducted using the KAPA SYBR FAST Universal qPCR kit (Kapa Biosystems, Boston, MA, USA) on a Thermal Cycler Dice system (TaKaRa). The specific primers employed included GAPDH (forward: 5′-TCTCTGCTCCTCCTGTTC-3′, reverse: 5′-CTCCGACCTTCACCTTCC-3′), CL-1 (forward: 5′-GGCAGATCCAGTGCAAATC-3′, reverse: 5′-TCTTCTGCACCTCATCGTCTT-3′), CL-4 (forward: 5′-GGCGTGGTGTTCCTGTTG-3′, reverse: 5′-AGCGGATTGTAGAAGTCTTGG-3′), ZO-1 (forward: 5′-CAGCCGGTCACGATCTCCT-3′, reverse: 5′-TCCGGAGACTGCCATTGC-3′), and Slug (forward: 5′-GAGCATACAGCCCCATCACT-3′, reverse: 5′-GGGTCTGAAAGCTTGGACTG-3′). GAPDH expression was consistent across samples and was used as the internal reference gen for normalization.

### 2.7. In Situ Loop Assay

Female C57BL/6 mice (10–12 weeks old) were purchased from SLC, Inc. (Shizuoka, Japan). They were housed under controlled conditions (23 ± 1.5 °C, 55% relative humidity) with a 12 h light/12 h dark cycle and had free access to standard rodent chow and water. All animal procedures were performed in accordance with Setsunan University guidelines and approved by the Ethical Use of Experimental Animals Committee (protocol codes K13-08 and K-14-11).

Under anesthesia, a midline abdominal incision was performed, and the jejunal lumen was initially washed with phosphate-buffered saline (PBS, pH 6.5). A 3 cm segment of jejunum was isolated by suture ligation of both ends. FITC-dextran (1.0 mg), with or without TCDD, was dissolved in 100 µL PBS (pH 6.5), was administered into the jejunal loop, after which the organs were carefully repositioned. Blood was collected from the caudal vena cava at specified time points, and the subsequent serum FITC-dextran concentrations were measured using a TriStar LB 941 microplate reader. The area under the plasma concentration–time curve from 0 to 6 h (AUC_0–6 h_) was determined using the non-compartmental trapezoidal method.

### 2.8. Western Blot Analysis of CL-4 Protein Expression

Caco-2 cells were lysed in buffer containing 20 mM Tris (pH 7.4), 150 mM NaCl, 1% SDS, and protease and phosphatase inhibitor cocktails, and subsequently sonicated for 1 min using 15-s cycles. Lysates were centrifuged at 18,000× *g* for 5 min. The supernatants were collected and stored at −80 °C until analysis.

Jejunal loops were washed with PBS (pH 6.5) and opened longitudinally. The tissue was then cut into ~5 mm fragments and washed with cold PBS (pH 7.4). Fragments were incubated in PBS containing 2 mM EDTA and 0.5 mM dithiothreitol for 30 min at 37 °C with gently shaking, and subsequently passed through a 70-µm mesh. Isolated cells were collected by centrifugation at 830× *g* for 5 min. These cells were then lysed in lysis buffer, sonicated, and centrifuged at 18,000× *g* for 5 min. The supernatants were stored at −80 °C until analysis.

Cell lysate protein content was quantified using a BCA protein assay kit (standardized with bovine serum albumin). Lysates were mixed with Laemmli sample buffer, boiled, and subjected to SDS-PAGE. Separated proteins were transferred onto PVDF membranes blocked with BlockAce (Yukijirushi Nyugyo Co., Tokyo, Japan) for 2 h at room temperature. Subsequently, membranes were incubated with primary antibodies against CL-1, CL-4 (Invitrogen, Carlsbad, CA, USA), Slug (Santa Cruz Biotechnology, Dallas, TX, USA), and β-actin (Wako Chemicals, Osaka, Japan) in Tris-buffed saline with 0.05% Tween 20 (TBS-T) for 2 h at room temperature. Membranes were then incubated with horseradish peroxidase-conjugated secondary antibodies (Merck Millipore, Darmstadt, Germany) in TBS-T for 1 h at room temperature, before immunoreactive signals were visualized by chemiluminescence (Nacalai Tesque) using a ChemiDoc imaging system (Bio-Rad Laboratories, Hercules, CA, USA).

### 2.9. Statistical Analysis

Data were analyzed by one-way analysis of variance (ANOVA) followed by Dunnett’s multiple comparison test. A *p*-value of less than 0.05 was considered statistically significant. Statistical analyses were performed using GraphPad Prism (v.10; GraphPad Software, San Diego, CA, USA).

## 3. Results

### 3.1. Screening for Environmental Pollutants with Disruptive Effects on Epithelial Barrier Function

**Figure 1:**
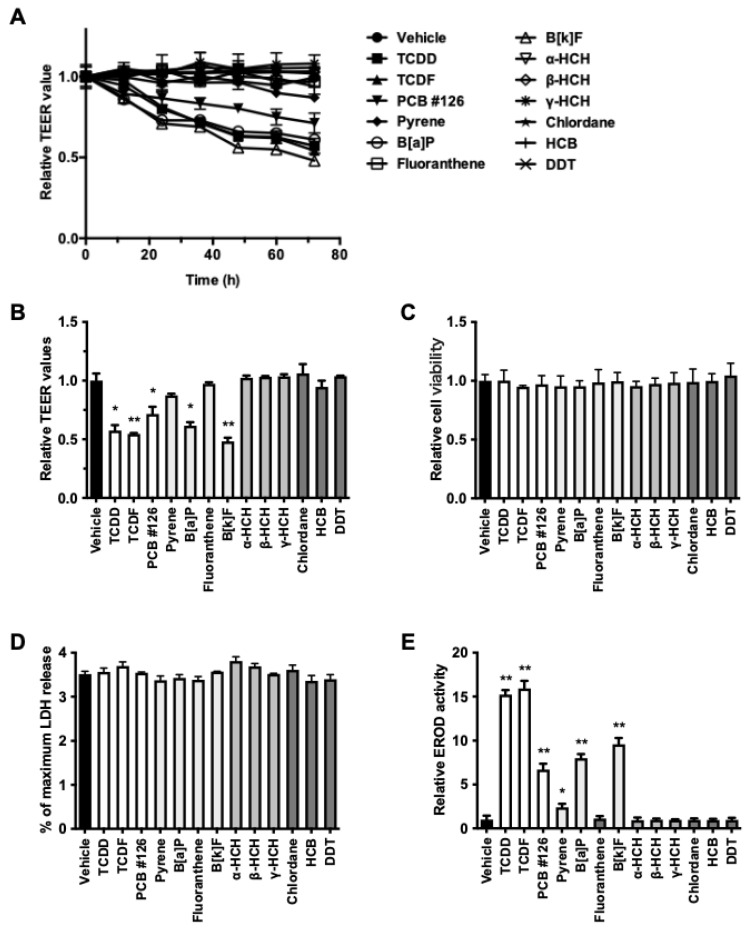
Environmental pollutants that disrupt barrier function. Differentiated Caco-2 cells in Transwell inserts (21 days) were stimulated with 100 nM TCDD, 100 nM TCDF, 100 nM PCB #126, 10 µM pyrene, 10 µM B[*a*]P, 10 µM fluoranthene, 10 µM B[*k*]F, 10 µM α-HCH, 10 µM β-HCH, 10 µM γ-HCH, 10 µM chlordane, 10 µM HCB, or 10 µM DDT. TEER values were measured over time (**A**) and quantified at 72 h (**B**). At 72 h, cytotoxicity was determined using WST-8 (**C**) and LDH release assays (**D**). EROD activity (**E**) was determined after 24 h of incubation. Values are shown as the mean ± SD (*n* = 5 per group). * *p* < 0.05, ** *p* < 0.01 vs. vehicle.

To determine whether environmental pollutants compromise intestinal epithelial barrier integrity, TEER was measured in polarized Caco-2 monolayers. TEER values progressively decreased following exposure to 2,3,7,8-TCDD, 2,3,7,8-tetrachlorodibenzofuran (TCDF), PCB #126, benzo[*a*]pyrene (B[*a*]P), and benzo[*k*]fluoranthene (B[*k*]F) (Figure 1A). After 72 h, these compounds reduced TEER by 42.6%, 45.4%, 28.5%, 38.4%, and 51.8%, respectively (Figure 1B). Pyrene and fluoranthene caused modest but statistically non-significant decreases in TEER, while α-, β-, and γ-hexachlorocyclohexane (HCH), chlordane, hexachlorobenzene (HCB), and dichlorodiphenyltrichloroethane (DDT) produced no appreciable effect. Notably, compounds that reduced TEER also induced ethoxyresorufin-*O*-deethylase (EROD) activity, suggesting a link to AhR activation (Figure 1E). Crucially, cell viability, as assessed by WST-8 and LDH release assays, remained unaffected (Figure 1C,D). However, treated cells exhibited morphological alterations, suggesting that these compounds alter cell structure rather than causing overt cytotoxicity.

### 3.2. Involvement of AhR in Pollutant-Induced Barrier Disruption

**Figure 2:**
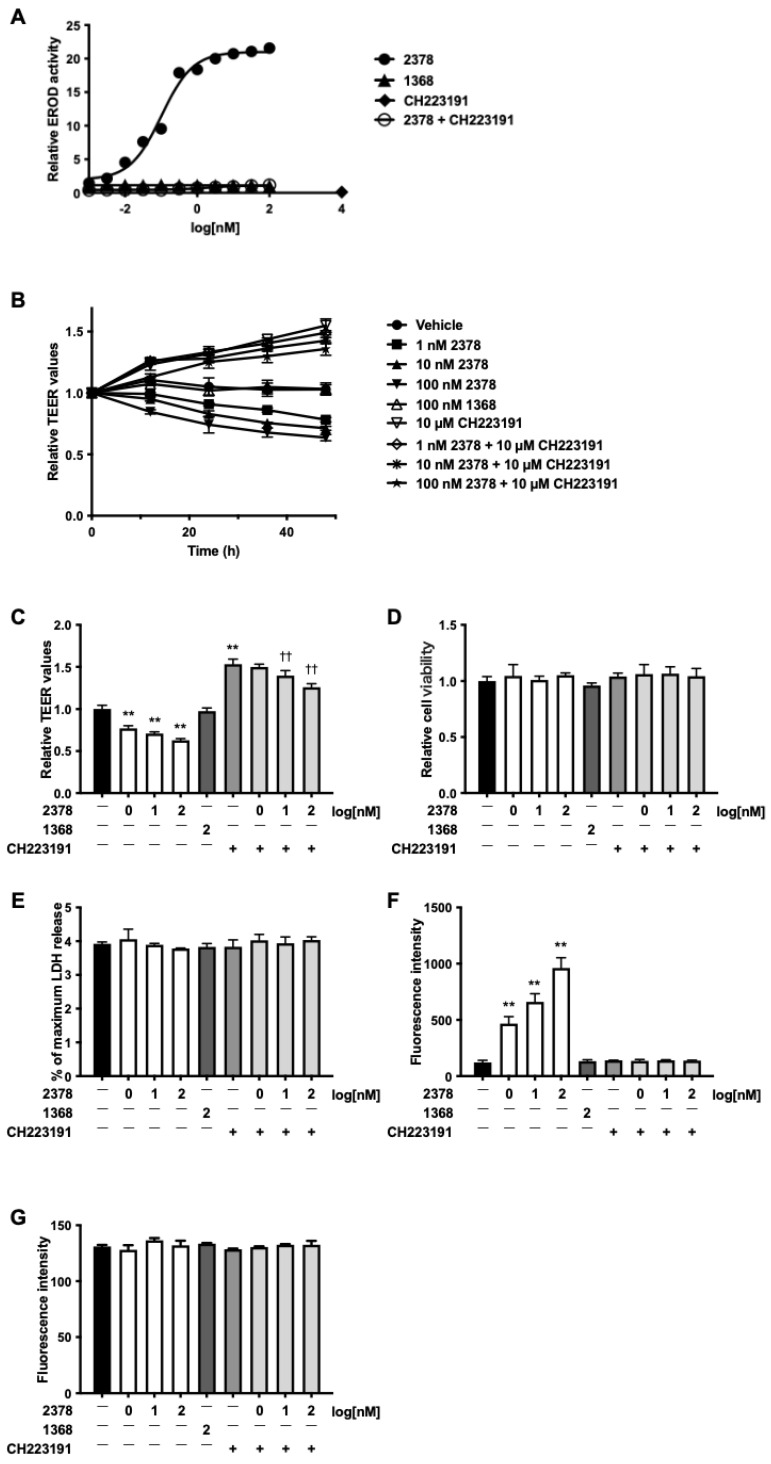
The AhR ligand disrupted barrier function. Monolayers of Caco-2 cells were stimulated with 2,3,7,8-TCDD or 1,3,6,8-TCDD for 24 h in the absence or presence of 10 µM CH223191. EROD activity was measured at 24 h (**A**). TEER values were monitored over time (**B**) and quantified at 48 h (**C**). At 48 h, cytotoxicity was determined using WST-8 (**D**), LDH release (**E**) assays. Paracellular flux assays were also conducted with FD-4 (**F**) and FD-10 (**G**). Values are shown as the mean ± SD (*n* = 5 per group). ** *p* < 0.01 vs. vehicle. †† *p* < 0.01 vs. CH223191-treated group.

Compounds associated with TEER reduction also elicited EROD activity (Figure 1E), suggesting that AhR activation may mediate this effect. Therefore, 2,3,7,8-TCDD was selected as a model ligand to clarify the role of AhR. In Caco-2 cells, 2,3,7,8-TCDD induced EROD activity in a concentration-dependent manner (EC_50_ = 0.102 nM), which was abolished by the AhR antagonist CH223191 (Figure 2A). In contrast, 1,3,6,8-TCDD failed to induce EROD activity. Similarly, 2,3,7,8-TCDD caused a concentration- and time-dependent reduction in TEER, with an approximately 40% decrease at 48 h following exposure to 10 nM, whereas 1,3,6,8-TCDD had no effect (Figure 2B,C). Importantly, CH223191 prevented the 2,3,7,8-TCDD-induced TEER reduction. Furthermore, neither congener affected cell viability (Figure 2D,E). To further characterize the barrier defect, paracellular flux of fluorescent dextrans was assessed. 2,3,7,8-TCDD increased the permeability of 4 kDa dextran in a dose-dependent manner, whereas the permeability of 10 kDa dextran remained unchanged (Figure 2F,G).

### 3.3. Effect on TJ-Associated Protein Expression

**Figure 3:**
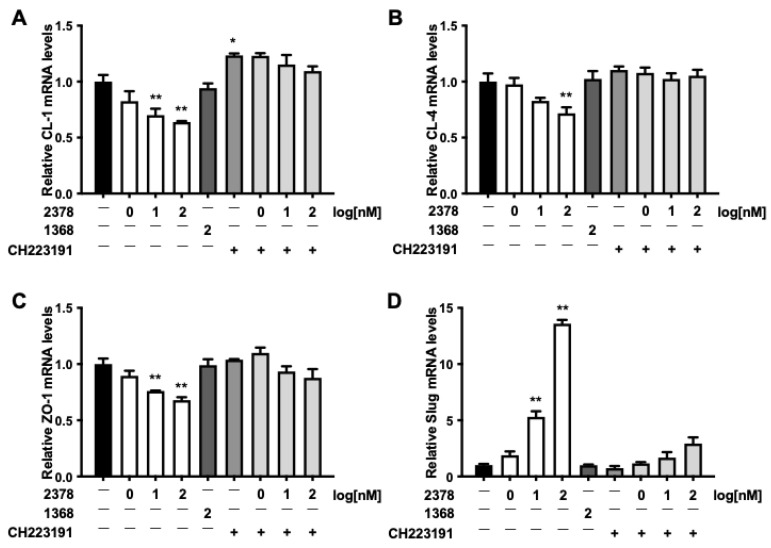

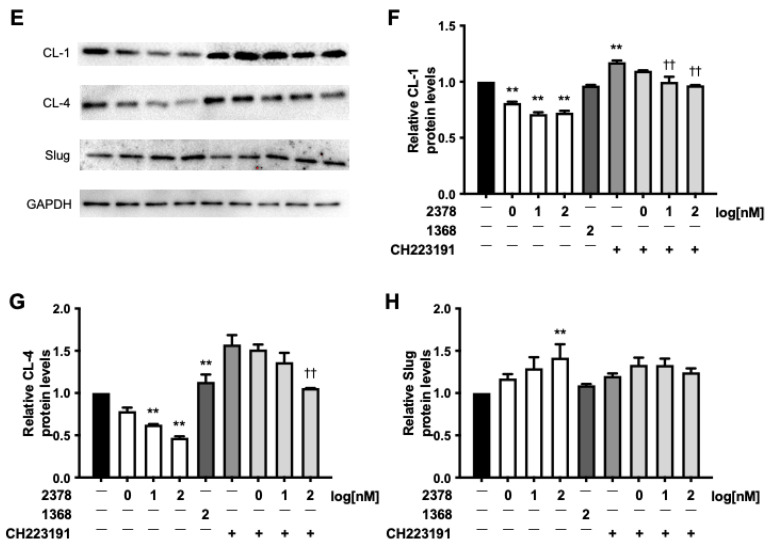
Expression of TJ-related protein. Monolayers of Caco-2 cells were stimulated with 2,3,7,8-TCDD or 1,3,6,8-TCDD for 48 h in the absence or presence of CH223191. After incubation, mRNA levels of CL-1 (**A**), CL-4 (**B**), ZO-1 (**C**), and Slug (**D**) were measured using quantitative real-time PCR. Protein expression was measured using Western blotting (**E**). The intensity of the CL-1 (**F**), CL-4 (**G**), and Slug (**H**) bands was quantified. Relative mRNA and protein levels are expressed as fold-induction relative to vehicle-treated cells. Values are shown as the mean ± SD (*n* = 5 per group). * *p* < 0.05, ** *p* < 0.01 vs. vehicle. †† *p* < vs. CH223191-treated group.

TJ proteins are the central determinants of intestinal barrier integrity, and thus changes in their expression or localization provide a mechanistic link between AhR activation by POPs and increased epithelial permeability. Treatment with 2,3,7,8-TCDD resulted in a concentration-dependent decrease in the mRNA levels of claudin-1 (CL-1), claudin-4 (CL-4), and zonula occludens-1 (ZO-1), with corresponding reductions of 36.1%, 28.3%, and 32.2%, respectively, observed at 100 nM (Figure 3A–C). Significantly, these effects were reversed by CH223191. Moreover, protein expression of CL-1 and CL-4 exhibited a similar pattern (Figure 3E,F). Conversely, expression of the transcription factor Slug was markedly upregulated by 2,3,7,8-TCDD in a concentration-dependent manner, and this effect was also attenuated by CH223191 (Figure 3D,G).

### 3.4. In Vivo Evidence of Intestinal Barrier Disruption

**Figure 4:**
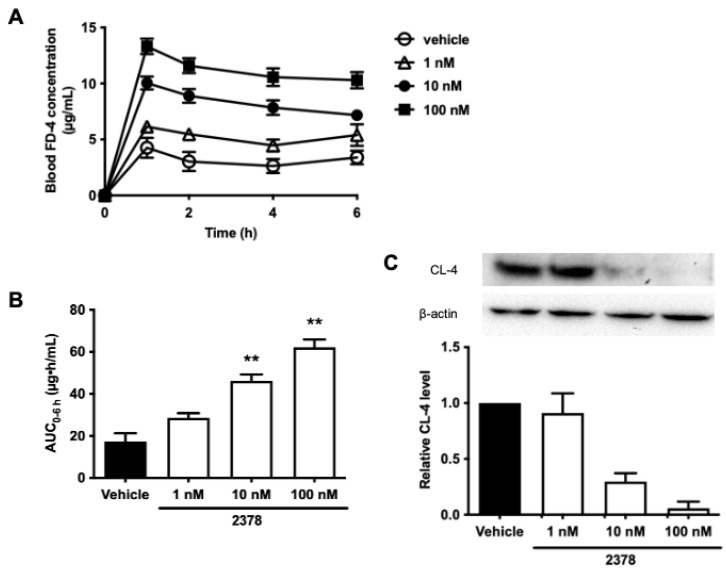
Blood transfer of FD-4 by AhR ligand-stimulation using an in situ loop. A mixture of 2,3,7,8-TCDD and FD-4 was administered to loops of mouse jejunum. The amount of FD-4 transferred into the blood over time was measured (**A**). The AUC was calculated up to 6 h (**B**). CL-4 expression in the jejunal loop incubated for 6 h was evaluated by Western blotting (**C**). Values are shown as the mean ± SEM (*n* = 10 per group). ** *p* < 0.01 vs. vehicle.

An in situ mouse jejunal loop model was therefore employed to validate the in vitro findings in vivo. Co-administration of 2,3,7,8-TCDD with FITC–dextran (4 kDa, FD-4) led to increased systemic absorption in a dose-dependent manner. The peak plasma FD-4 concentration at 1 h was 4.3 µg/mL in controls, versus compared with 6.1, 10.0, and 13.3 µg/mL following treatment with 1, 10, and 100 nM 2,3,7,8-TCDD, respectively (Figure 4A,B). The AUC_0–6 h_ also increased accordingly. Crucially, expression of CL-4 in isolated jejunal epithelial cells was decreased in 2,3,7,8-TCDD-treated groups, consistent with the in vitro observations (Figure 4C). Taken together, these findings demonstrate that exposure to AhR ligands compromises intestinal barrier integrity via suppression of CL-4.

## 4. Discussion

We demonstrate here that POPs possessing AhR binding capacity—including 2,3,7,8-TCDD, TCDF, PCB #126, B[*a*]P, and B[*k*]F—impair intestinal epithelial TJ integrity, resulting in functional barrier loss both in vitro and in vivo. Importantly, TEER reduction occurred at concentrations that did not elicit overt cytotoxicity, thereby indicating modulation of junctional regulation rather than cell death per se.

Our mechanistic data identify AhR as a central mediator of pollutant-induced barrier dysfunction. TEER-reducing pollutants exhibited EROD activity, consistent with AhR activation. The high-affinity ligand 2,3,7,8-TCDD induced concentration-dependent decreases in TEER and enhanced dextran permeability, both of which were abolished by the selective AhR antagonist CH223191. Comparisons with the non-binding congener 1,3,6,8-TCDD further demonstrate the ligand-specificity of the response. Collectively, these findings indicate that AhR activation by persistent, high-affinity ligands can drive TJ remodeling and barrier compromise, which is consistent with prior in vitro and in vivo reports linking AhR hyperactivation to intestinal leakiness [16,18,20].

At the molecular level, we observed the preferential downregulation of CL-4 together with reductions in CL-1 and ZO-1, accompanied by upregulation of the transcriptional repressor Slug. The selective suppression of CL-4 is particularly noteworthy, as its sealing role in regulating paracellular permeability plausibly explains the increased small-molecule flux observed in this study. These results are consistent with a model in which persistent AhR agonists activate a Slug-dependent transcriptional program that represses CL-4 and destabilizes TJ assembly.

Although multiple POPs reduced TEER and activated AhR, mechanistic analyses (CL-4 downregulation and Slug upregulation) were performed only for TCDD. Therefore, it remains uncertain whether all POPs impair the barrier through the same molecular pathway. Differences in ligand affinity, persistence, and structural features may produce distinct downstream signaling effects, underscoring the need for further studies to determine whether the TCDD mechanism is generalizable across pollutant classes.

Our results also demonstrate that barrier dysfunction can occur independently of cytotoxicity and identify specific TJ components affected by TCDD. However, a more comprehensive mapping of junctional proteins targeted by other POPs—and confirmation of these mechanisms in vivo—remains necessary. Additionally, since Caco-2 cells are derived from colon adenocarcinoma, they may not fully recapitulate the physiological properties of the small intestinal (jejunal) epithelium. Although the in situ mouse jejunal loop model validated key findings, data were limited to one intestinal segment and species, warranting cautious extrapolation to the entire human gut and to other pollutants.

AhR activation increased permeability to FD-4 but not to FD-10, indicating that barrier impairment primarily affects small molecules. However, systemic absorption of larger or clinically relevant macromolecules, such as endotoxins or dietary antigens, was not evaluated. Thus, while FD-4 permeability confirms TJ disruption, additional studies are required to assess the pathological consequences of POPs-induced “leaky gut”.

Comparative literature supports a ligand-dependent duality of AhR signaling in the gut. Physiological or rapidly metabolized dietary and microbial AhR ligands—such as indoles and tryptophan derivatives—generally promote epithelial homeostasis and mucosal defense [21], whereas high-affinity, persistent xenobiotic ligands such as TCDD trigger prolonged receptor activation leading to dysregulated gene expression and inflammation [22,23,24]. This ligand-quality dependence reconciles the apparently opposing roles of AhR in mucosal protection versus pollutant toxicity.

Emerging evidence further suggests that pollutant-induced dysbiosis may amplify these effects. POP exposure alters gut microbial composition and reduces the generation of endogenous, low-affinity AhR ligands, thereby lowering the protective “AhR tone” of the intestine [25,26,27]. This microbial contribution may create a two-hit scenario in which direct activation of AhR by high-affinity POPs combines with the loss of microbiota-derived protective ligands. Such synergy could explain why complex real-world exposures often produce disproportionate intestinal and systemic effects.

Our findings indicate that intestinal barrier integrity—particularly CL-4 expression—may serve as a sensitive mechanistic endpoint for assessing subclinical toxicity of environmental contaminants. Single-gene biomarkers are insufficient; instead, CL-4 should be integrated into a multiparametric biomarker panel that includes TEER, size-selective permeability assays, AhR activation indices (such as EROD and CYP1A1 expression), and quantification of microbiome-derived ligands [28]. Such a composite approach would enhance both the mechanistic specificity and predictive value of barrier-based toxicity testing.

Finally, the ligand-dependent duality of AhR signaling suggests both hazard and therapeutic potential. Persistent, high-affinity AhR agonists such as TCDD represent a clear risk for epithelial barrier dysfunction and systemic endotoxemia, whereas controlled activation by dietary or pharmacological ligands may enhance barrier resilience and mucosal immunity [21]. Clarifying this balance will require studies integrating ligand affinity, metabolic persistence, microbiome status, and host AhR polymorphisms under chronic low-dose exposure scenarios relevant to human populations.

## 5. Conclusions

In summary, our integrated data reveal that AhR-binding POPs compromise intestinal barrier integrity through TJ remodeling characterized by CL-4 downregulation and Slug upregulation. These mechanistic insights—particularly when considered alongside the emerging understanding of microbiome–AhR interactions—highlight the need to incorporate epithelial barrier endpoints into environmental health risk assessment. Furthermore, these findings provide a basis for developing AhR-targeted interventions to preserve gut homeostasis under pollutant exposure.

## Data Availability

The data presented in this study are available on request from the corresponding author.

## Abbreviations

The following abbreviations are used in this manuscript:

1368	1,3,6,8-TCDD
2378	2,3,7,8-TCDD
AhR	aryl hydrocarbon receptor
B[*a*]P	benzo[*a*]pyrene
B[*k*]F	benzo[*k*]fluoranthene
CL-1	claudin-1
CL-4	claudin-4
CYP	cytochrome P450
DDT	dichlorodiphenyltrichloroethane
EROD	ethoxyresorufin-*O*-deethylase
HCB	hexachlorobenzene
HCH	hexachlorocyclohexane
LDH	lactate dehydrogenase
POPs	persistent organic pollutants
TCDD	tetrachlorodibenzo-*p*-dioxin
TEER	transepithelial electrical resistance
ZO-1	zonula occludens-1
TCDF	2,3,7,8-tetrachlorodibenzofuran
